# Chronic stress disrupts neural coherence between cortico-limbic structures

**DOI:** 10.3389/fncir.2013.00010

**Published:** 2013-02-06

**Authors:** João Filipe Oliveira, Nuno Sérgio Dias, Mariana Correia, Filipa Gama-Pereira, Vanessa Morais Sardinha, Ana Lima, Ana Filipa Oliveira, Luís Ricardo Jacinto, Daniela Silva Ferreira, Ana Maria Silva, Joana Santos Reis, João José Cerqueira, Nuno Sousa

**Affiliations:** ^1^School of Health Sciences, Life and Health Sciences Research Institute (ICVS), University of MinhoBraga, Portugal; ^2^ICVS/3B's - PT Government Associate LaboratoryBraga/Guimarães, Portugal; ^3^DIGARC, Polytechnic Institute of Cávado and AveBarcelos, Portugal; ^4^Department of Industrial Electronics, University of Minho - Campus de AzurémGuimarães, Portugal

**Keywords:** chronic stress, coherence, power spectrum, hippocampus, prefrontal cortex

## Abstract

Chronic stress impairs cognitive function, namely on tasks that rely on the integrity of cortico-limbic networks. To unravel the functional impact of progressive stress in cortico-limbic networks we measured neural activity and spectral coherences between the ventral hippocampus (vHIP) and the medial prefrontal cortex (mPFC) in rats subjected to short term stress (STS) and chronic unpredictable stress (CUS). CUS exposure consistently disrupted the spectral coherence between both areas for a wide range of frequencies, whereas STS exposure failed to trigger such effect. The chronic stress-induced coherence decrease correlated inversely with the vHIP power spectrum, but not with the mPFC power spectrum, which supports the view that hippocampal dysfunction is the primary event after stress exposure. Importantly, we additionally show that the variations in vHIP-to-mPFC coherence and power spectrum in the vHIP correlated with stress-induced behavioral deficits in a spatial reference memory task. Altogether, these findings result in an innovative readout to measure, and follow, the functional events that underlie the stress-induced reference memory impairments.

## Introduction

Stress is a constant in the daily life of the modern societies. Each subject is constantly challenged and threatened by a large variety of unpredicted events. Under stressful situations, a primary response is set up, in order to restore homeostasis and promote behavioral adaptation; however, prolonged stress exposure may trigger maladaptive responses that lead to severe manifestations such as learning and memory deficits or anxious and depressive-like behavior (Popoli et al., 2011; Sousa and Almeida, 2012). Whereas hippocampal structural damage and impaired plasticity were initially recognized to underlie these manifestations (Sousa et al., 2000), subsequent studies demonstrated that the medial prefrontal cortex (mPFC), an area intimately related with working memory processes and cognitive stimuli integration, is also a key target of chronic stress (Cerqueira et al., 2007a,b; Liston et al., 2009). At a functional level, such neuronal compromise was correlated with a strong impairment of plasticity in the hippocampus-PFC pathway (Cerqueira et al., 2007a).

Although mechanisms of plasticity are generally accepted as readouts of interregional connections, much information on neuronal dynamics is lost due to the supra-physiological protocols typically used. Since previous data suggests that chronic unpredictable stress (CUS) triggers disconnections in specific brain circuits, additional measures such as power spectra and phase coherence of local field potentials (LFPs) under stressful conditions urge to be assessed. LFPs reproduce summated individual conductance and synaptic inputs of networks composed by ensembles of firing neurons and surrounding glia, and therefore are an excellent readout of network dynamics (Buzsáki, 2006, 2010; Perea et al., 2009; Jia et al., 2011). LFPs reflect the temporal pattern of activity that acts on local networks that are directly connected (Varela et al., 2001); importantly, the ventral hippocampus (vHIP) and the prelimbic subfield of the medial PFC are linked by monosynaptic connection (Jay and Witter, 1991; Thierry et al., 2000; Tierney et al., 2004). Moreover, phase coherence between the hippocampus and cortex was proposed to correlate with acquisition of information and memory formation (Buzsáki, 1996; Popa et al., 2010; Fell and Axmacher, 2011). In fact, previous studies using maze-based paradigms to test different types of memory, reveal that rats displayed increased theta phase coherence between the PFC and the HIP by the time they take a decision based in a previous experience (Jones and Wilson, 2005; Benchenane et al., 2010); in addition, hippocampal theta rhythms were shown to synchronize with PFC single cell (Siapas et al., 2005) and field activities (Hyman et al., 2011). Beta coherence between hippocampus and mPFC was also shown to be necessary for effective communication during visual object processing (Sehatpour et al., 2008). Interestingly, impaired coherence in the hippocampus-mPFC was observed after loss of PFC function (Brockmann et al., 2011) and in models of schizophrenia (Sigurdsson et al., 2010).

Based on these assumptions we hypothesize that stress-induced deleterious effects on the function of the PFC and hippocampus may be in a large extent due to alterations in phase coherence between these areas. The present study tested this hypothesis by recording neural activity simultaneously from the mPFC and vHIP in rats exposed to short-term and chronic stress; the use of different periods of exposure to stress provided an insight of the temporal dynamics of the onset of stress-induced changes. Power spectrum densities and coherence were also analyzed, and correlations between them were studied and compared with classical long-term potentiation measurements. In addition, in a separate set of rats, variations in coherence and power spectrum were correlated with behavioral performance.

## Results

### Stressed rats display increased power spectrum densities in the vHIP and mPFC

Power spectra translate the amplitude of the signals recorded in a brain region on the frequency domain. power spectrum density (PSD) analysis of the recorded LFPs from the mPFC and vHIP of all studied rats allowed the thorough characterization of power activity in a wide range of frequencies (Delta, 1–4 Hz; Theta, 4–12 Hz; Beta, 12–20 Hz; Low Gamma, 20–40 Hz; High Gamma, 40–90 Hz) for those regions at a basal state.

**Figure 1:**
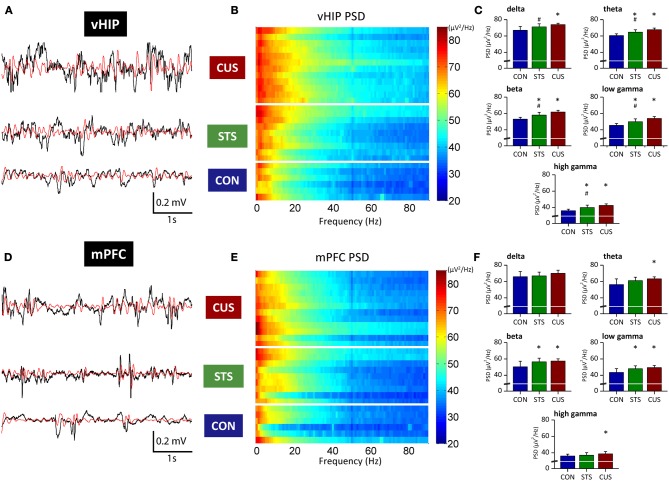
**Stressed rats show increased PSD in multiple frequency bands both in the ventral hippocampus and medial PFC.** Representative traces of raw data (black line) recorded simultaneously from the vHIP **(A)** and mPFC **(D)** of a rat of each group; the red line represents theta filtered component, as example. Power Spectral Density (PSD) values of the vHIP **(B)** recordings and the mPFC **(E)** recordings, for controls (CON), short-term stress (STS), and chronic unpredictable stress (CUS); each horizontal line in the Y-axis represents the spectrogram of an individual rat. Group comparison of the PSD values from vHIP **(C)** and mPFC **(F)** in the delta (1–4 Hz), theta (4–12 Hz), beta (12–20 Hz), low gamma (20–40 Hz), and high gamma (40–90 Hz) frequency bands. ^*^Statistically different from CON, *p* < 0.05; ^#^Statistically different from CUS, *p* < 0.05; error bars represent SD.

Regarding the function of the vHIP, short term stress (STS) triggered an increase of PSD in the theta, beta, low gamma and high gamma frequencies, while in the delta band no significant variation to controls (CON) was recorded (Figures 1A–C; see Table 1 for statistical values). Importantly, CUS exposure-induced a persistent increase in PSD in all frequency bands analyzed which was always higher than controls and STS (Figures 1A–C; see Table 1).

**Table 1:**
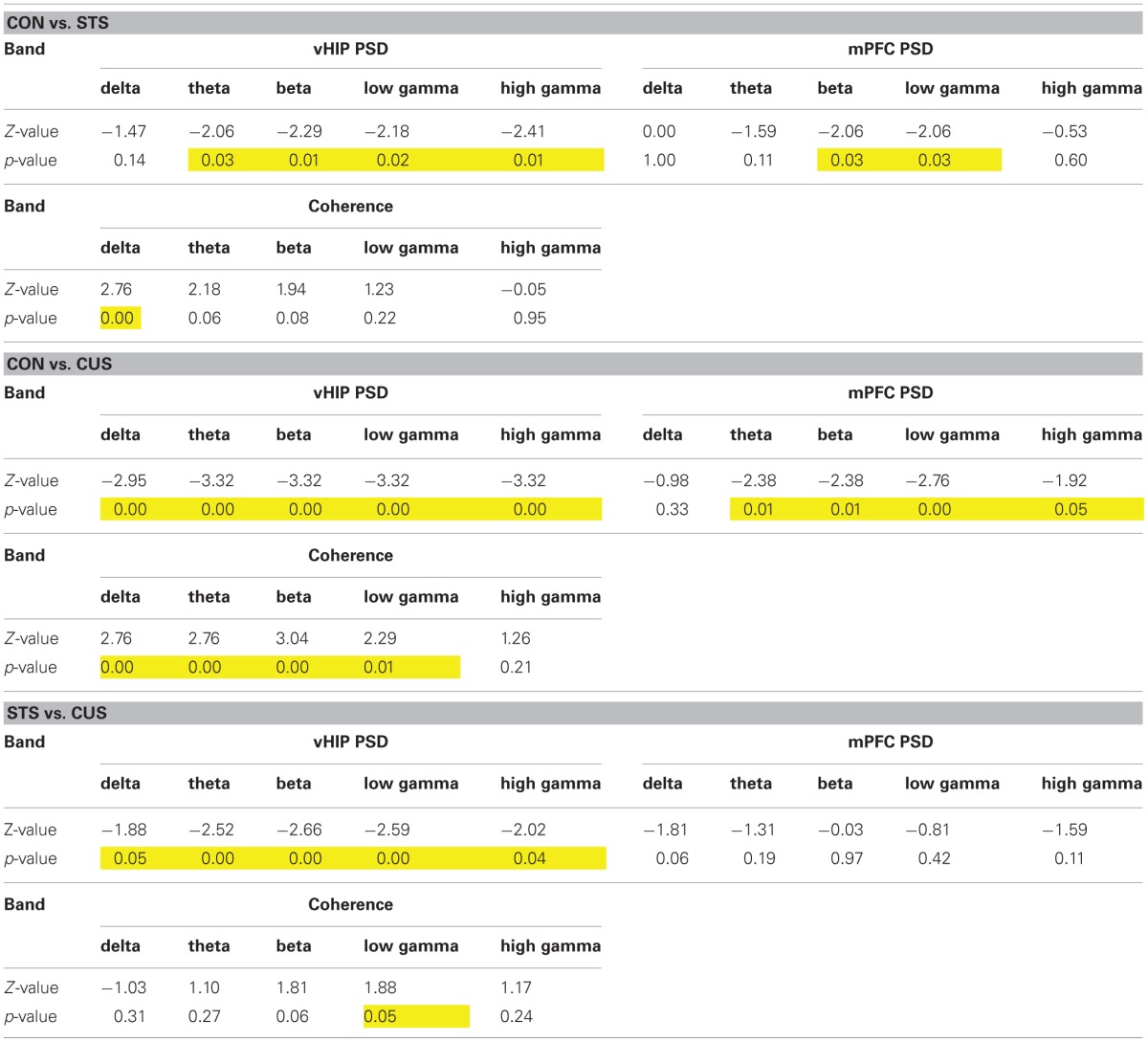
**Statistical values of the group comparisons of PSD and coherence for each frequency band in the first set of animals**.

**Figure 2:**
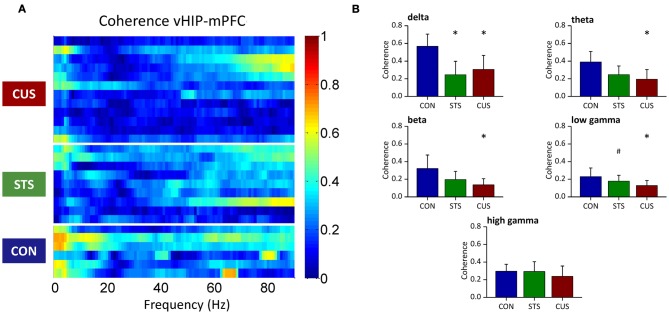
**Chronic stress decreases the coherence between the ventral hippocampus (vHIP) and the medial prefrontal cortex (mPFC). (A)** Spectral coherence of controls (CON), short term stress (STS), and chronic unpredictable stress (CUS); each horizontal line in the Y-axis represents the spectrogram of an individual rat **(B)** Group comparison of the coherence values between mPFC and vHIP for delta (1–4 Hz), theta (4–12 Hz), beta (12–20 Hz), low gamma (20–40 Hz), and high gamma (40–90 Hz) frequency bands. ^*^Statistically different from CON, *p* < 0.05; ^#^Statistically different from CUS, *p* < 0.05; error bars represent SD.

*Each table represents the Z- and p-values of pairwise comparisons of vHIP PSD, mPFC PSD, and coherence values in the control (CON), short-term stress (STS), and chronic unpredictable stress (CUS) groups. These values correspond to the graphs presented in Figures 1 and 2. Comparisons made by Mann–Whitney test; statistical significance is highlighted in yellow (p ≤ 0.05)*.

In contrast, PSD in the mPFC was only affected by the exposure to STS in beta and low gamma frequencies when compared to control rats (Figures 1D–F; Table 1); however, exposure to CUS triggered an increase in the PSD in the theta, beta and low gamma and high gamma frequency bands (Figures 1D–F; Table 1).

### Stress decreases spectral coherence between the vHIP and mPFC

Coherence between brain regions measures the matching of temporal structure in signals recorded from those regions. The temporal structure, or phase, is a powerful measure since it does not depend on signal amplitude and two signals are said to be synchronous if their rhythms' phase match (Varela et al., 2001). Coherence analysis of the signals obtained simultaneously from the vHIP and mPFC was used to study phase coherence between these regions of the brain (Figure 2). The exposure of the rats to STS caused a significant decrease of phase coherence in the delta band when compared to the observed in controls (CON) (Figure 2A for individual analysis, Figure 2B for group comparison by frequency band; statistic results on Table 1). Interestingly, exposure to CUS-induced a pronounced decrease in phase coherence when comparing to CON rats in the delta, theta, alpha, beta and low gamma bands, and in the low gamma band when compared to STS; in the high gamma band, coherence levels of CUS subjects were statistically undistinguishable from those obtained in the CON rats (Figures 2A,B; Table 1) and therefore further analysis was focused on the data observed for frequencies below 40 Hz.

### Stress-induced loss of coherence correlates with increased PSD in the vHIP

**Figure 3:**
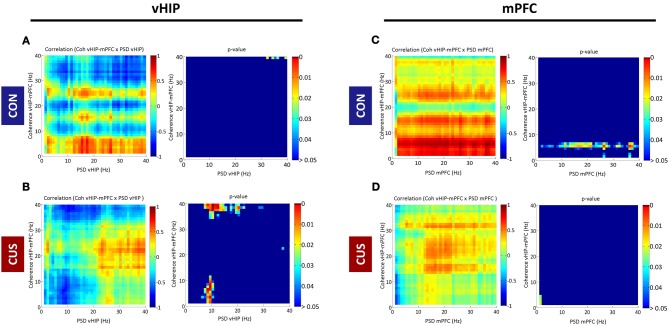
**Correlations between spectral coherence and power spectral densities in ventral hippocampus (vHIP) and medial prefrontal cortex (mPFC).** The graphs present Pearson values for correlations between vHIP-mPFC spectral coherence and vHIP **(A,B)** or mPFC **(C,D)** power spectral densities and respective *p*-values against null hypothesis (corr = 0), for CON and CUS rats (**A,C** and **B,D**, respectively). For each brain region, statistically significant correlations are represented in the right panel by color above dark blue (*p* < 0.05); the direction of the correlation, positive or negative, is given in the left panel by the color code (green to red, positive correlation; green to blue, negative correlation).

In order to disclose whether stress affects the relationship between power activity and coherence observed in the vHIP and mPFC of CON rats, a correlation between both measures was explored (Figure 3).

PSD recorded from the vHIP of CON rats did not correlate significantly with vHIP-mPFC coherence in the range of frequency bands analyzed (0–40Hz; CON in Figures 3A,B). Exposure to stress triggers a negative correlation between theta vHIP-PSD and delta-theta and low gamma coherence which links the observations in Figures 1 and 2, where coherence decreases as the power increases in vHIP as a result of exposure to stress. Such negative correlations are absent between vHIP-mPFC coherence and PSD recorded from the mPFC of stressed animals suggesting that these two measures are not linked. Interestingly, CON rats show a clear positive correlation between theta coherence and all frequencies in mPFC PSD which seems to be disrupted by the chronic exposure to stress (Figures 3C,D).

### Stress exposure impaired LTP induction in the hippocampal-mPFC link

**Figure A1:**
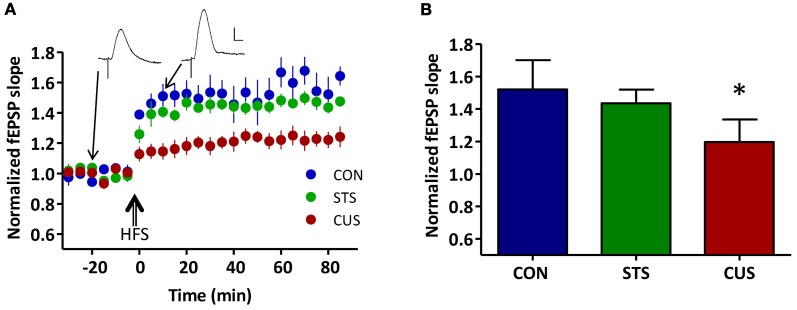
**Chronic unpredictable stress decreases synaptic plasticity between the ventral hippocampus (vHIP) and the medial prefrontal cortex (mPFC). (A)** Time course of LTP induction after high frequency stimulation (HFS); each circle represents the average of 10 normalized fEPSP slopes with SEM; three groups are depicted: controls (CON, blue), short term stress (STS, green) and chronic unpredictable stress (CUS, red); (inset) representative recordings of fEPSPs pre- and post-HFS (scale bar: 1 mV; 5 ms) of a CON rat; **(B)** Normalized values of LTP averaged for the three groups of rats: CON, STS, and CUS; ^*^statistically different from controls, *p* < 0.05; error bars represent SD.

To compare the above described results with classical electrophysiological measures of synaptic plasticity, we have analyzed the ability to induce LTP in this neuronal connection. When subjected to high frequency stimulation (HFS) in the vHIP, the slope of evoked PSPs recorded in the mPFC of CON rats increased about 52.1 ± 9.0% and remained elevated for at least 90 min, in the form of long term potentiation (LTP; as a measure of neural plasticity between the two regions; Figure A1A). The CUS rats displayed the previously described impairment of LTP between the vHIP and mPFC (CUS, 19.6 ± 4.7%; *p* < 0.05) (Cerqueira et al., 2007a), while the STS rats maintained the LTP values when compared to the CON rat (STS, 43.5 ± 3.5%; *p* > 0.05; Figure A1B).

### Behavioral performance correlates with variations in coherence and power spectrum density

An independent set of control and chronically stressed rats was tested for cognitive function in order to assess an eventual link between the chronic stress-induced coherence impairment and power increase observed in the first set of rats and the previously described cognitive impairments triggered by the chronic stress protocols described in the literature (Cerqueira et al., 2007a; Dias-Ferreira et al., 2009). The analysis of corticosterone levels as a measure of efficacy of the stress protocol confirmed that the CUS protocol induced a chronic stress state in the stressed rats (CON, 48.2 ± 5.9 ng/ml; CUS, 414.8 ± 41.5 ng/ml; *p* = 0.002).

**Figure 4:**
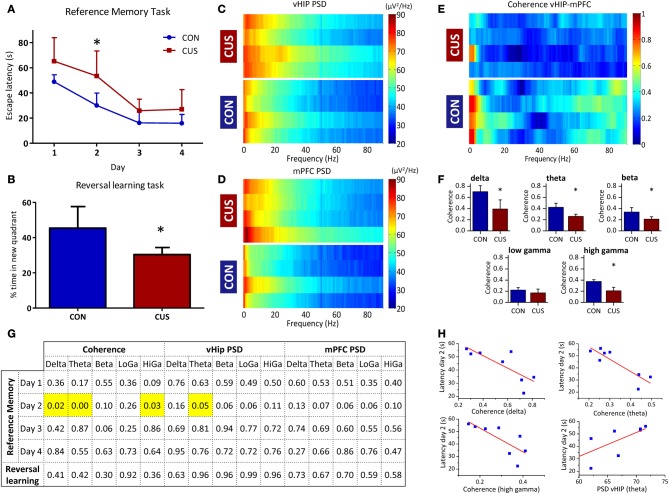
**CUS-induced changes in PSD and coherence correlate with impairments in cognitive performance. (A,B)** Cognitive performance of the studied rats in water maze based tests for spatial reference memory and behavioral flexibility; **(A)** Learning curves of the reference memory task of control (CON) and chronically stressed (CUS) rats. **(B)** Average trial time in the new quadrant given as a percentage of the total escape latency for the reversal learning task. **(C–F)** Analysis of the LFP signals recorded in the vHIP and mPFC of the CON and CUS rats; Power Spectral Density (PSD) values of the vHIP **(C)** recordings and the mPFC **(D)** recordings, for controls (CON) and chronically stressed (CUS); **(E)** Spectral coherence of controls (CON) and chronic unpredictable stress (CUS); in **(C–E)** each horizontal line in the Y-axis represents the spectrogram of an individual rat; **(F)** Group comparison of the coherence values between mPFC and vHIP for delta (1–4 Hz), theta (4–12 Hz), beta (12–20 Hz), low gamma (20–40 Hz), and high gamma (40–90 Hz) frequency bands. **(G,H)** Correlation between behavior and electrophysiological performances of the recorded rats; **(G)** *p*-values for Pearson correlations between behavior and electrophysiological performances for each rat; significant correlations highlighted in yellow; **(H)** Correlation plot for each significant correlation observed in G. ^*^Statistically different from CON, *p* < 0.05; error bars represent SD.

**Figure A2:**
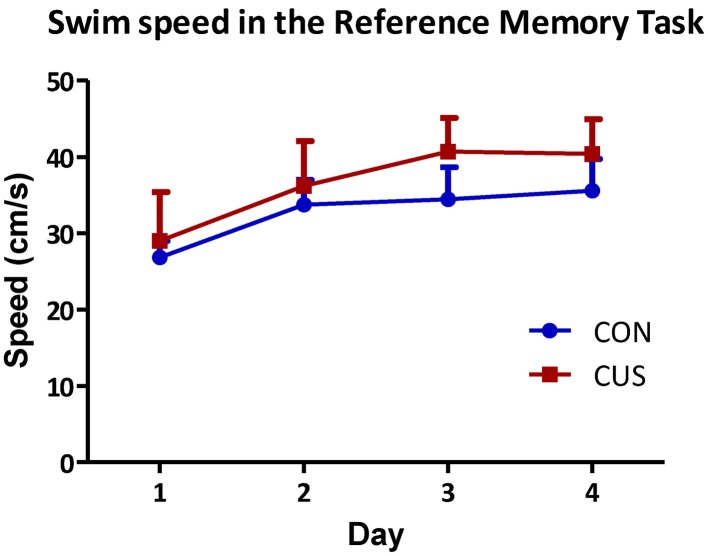
**Chronic stress exposure does not induce locomotor deficits**. Average swimming speeds of CON (blue) and CUS (red) rats during the 4 days spatial reference memory task. No differences are observed between groups in each day (*p* > 0.05); error bars represent SD.

Chronic stress triggered deficits in the spatial reference memory task, since CUS rats swim longer that controls to find the platform and the learning curves of CON and CUS are significantly different (Figure 4A; *p* = 0.02). *Post-hoc* analysis comparisons revealed significant impairments in reference memory in CUS rats on day 2 (Figure 4A; day 2, *t*-value = 3.126, *p* < 0.05; day 1, *t*-value = 2.178; day 3, *t*-value = 1.285; day 4, *t*-value = 1.480; *p* > 0.05). Importantly, the analysis of swimming speed of each group reported that both CON and CUS rats swim at a same speed in the water maze (Figure A2; *p* > 0.05) and *post-hoc* analysis sustained this observation for each day (day 1, *t*-value = 0.81; day 2, *t*-value = 0.93; day 3, *t*-value = 2.38; day 4, *t*-value = 1.83; *p* > 0.05), excluding any locomotor deficit due to the chronic stress treatment. Additionally, chronic stress triggered a behavioral flexibility impairment, since CUS rats spent less time in the new quadrant that control rats (Figure 4B; *p* = 0.04) in the reversal learning task of the Morris Water Maze, in agreement with data previously reported (Cerqueira et al., 2007a).

**Figure A3:**
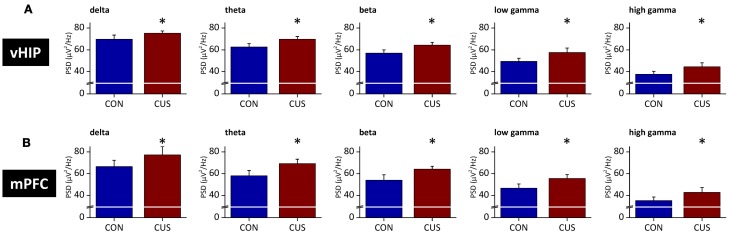
**Stressed rats show increased PSD in multiple frequency bands both in the ventral hippocampus and medial PFC in the second set of rats.** Group comparison of the power spectral density (PSD) values from vHIP **(A)** and mPFC **(B)** in the delta (1–4 Hz), theta (4–12 Hz), beta (12–20 Hz), low gamma (20–40 Hz) and high gamma (40–90 Hz) frequency bands. ^*^Statistically different from CON, *p* < 0.05; error bars represent SD.

Again, we confirmed in this additional set of rats that CUS triggered a general increase in power recorded in the vHIP when compared to the CON rats (Figures 4C, A3A; Table 2). Similarly, the CUS rats displayed an increased PSD in the mPFC when compared to the CON rats (Figures 4D, Figure A3B; Table 2). Regarding the coherence between the vHIP and mPFC (Figure 4E), the stressed rats displayed a general decrease of coherence in a wide range of frequency bands when compared to controls, except for the low gamma band, where a similar tendency was observed although not significant (Figure 4F; Table 2). These results were in accordance with the data recorded for the first set of rats, sustaining a profound affection of the network after chronic exposure to unpredictable stressors.

**Table 2:**
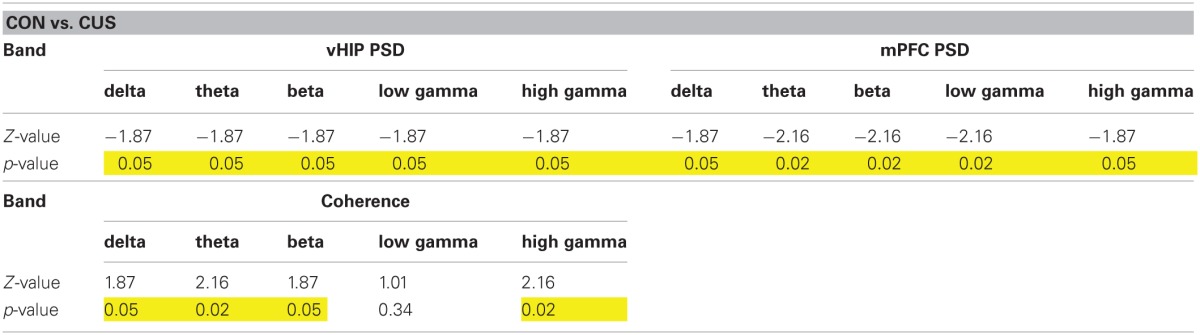
**Statistical values of the group comparisons of PSD and coherence for each frequency band in the second set of animals**.

*Each table represents the Z- and p-values of pairwise comparisons of vHIP PSD, mPFC PSD, and coherence values in the control (CON) and chronic unpredictable stress (CUS) groups. These values correspond to the graphs presented in Figure 4. Comparisons made by Mann–Whitney test; statistical significance is highlighted in yellow (p ≤ 0.05)*.

Subsequently, we searched for pairwise correlations among cognitive performance in each day of the spatial reference memory task, cognitive performance in the reversal learning task, coherence between the mPFC and vHIP and power spectra in these brain regions (Figures 4G,H). Data shows that the different performance of stressed and control rats on the second day of the reference memory task inversely correlates with the value of coherence measured between the vHIP and mPFC for each of those rats (Figure 4H) in the delta (*r* = −0.79), theta (*r* = −0.88) and high gamma (*r* = −0.77) bands, meaning that the coherence between the two regions is crucial for the good performance in this task. Additionally, we observed that the increase in theta power in the vHIP directly correlates with higher escape latencies in the second day of test (Figure 4H; *r* = 0.71), disclosing a link between stress-triggered increase in theta vHIP power and worse performance in this task by CUS rats.

## Discussion

Exposure to CUS was previously shown to induce deleterious effects at a morphological level in the hippocampus (Sousa et al., 2000) and mPFC (Cerqueira et al., 2007b), which were suggested to underlie the deficits observed in behavior paradigms that rely on those areas in rodents (Cerqueira et al., 2007a; Dias-Ferreira et al., 2009) and in humans (Soares et al., 2012). In this work, using anesthetized rats, we show an unequivocal decrease in phase coherence in stressed subjects when compared with their non-stressed rats. Additionally, PSD extracted from LFPs recorded in the mPFC of the CUS subjects increased significantly between 4 and 40 Hz, while a global increase of PSD in the vHIP was observed for both STS and CUS rats. We show finally that the decrease in delta, theta and high gamma coherence and the increase in theta power in the vHIP correlate with the worse performance in the reference memory task of the Morris Water Maze.

The impact of stress in PSD and coherence between interconnected brain regions is currently under scrutiny. A recent study reported a decrease in coherence around 4 Hz between both areas but no significant changes in PSD (Lee et al., 2011). The present data is quite distinct to the findings of that study. Although both sets of data report to the connection between the HIP and PFC, differences in experimental conditions may explain these apparently discrepant findings. First, in the present study we have recorded from a more ventral hippocampal region than the one recorded in the Lee et al. study; this is very relevant, since our placement was chosen based on evidence that the monosynaptic hippocampus-PFC connection originates in the vHIP (Jay and Witter, 1991). Moreover, it has been shown that the electrophysiological response of the vHIP to stress is remarkably different from the one observed in more dorsal regions (Maggio and Segal, 2009). The second methodological aspect that deserves merit to be highlighted is that the stress paradigms used in both studies were also different. While Lee et al. (2011) used a repeated stress paradigm, the unpredictability of stressor presentation of the CUS protocol used in our work was shown to be crucial for the stress-consequence severity in the neural regions affected (Maier and Watkins, 2005; Amat et al., 2006); this might also justify the severity of effects observed in our data.

CUS rats presented significant increases of PSD, when compared to non-stressed rats, in both vHIP and mPFC. Interestingly, the present results show that STS also increase PSD, but more extensively in the vHIP. Since power activity translates the amplitude of the signals recorded in a brain region for a given frequency, which in turn represents the extent of neuron recruitment to fire on a particular rhythm, one may conclude that exposure to stress caused an increase in neuronal activity, more pronounced in the vHIP, that progresses with time also to other brain regions, such as the mPFC. This temporal sequence has been previously proposed by our behavioral data (Cerqueira et al., 2007a) and is of great relevance to understand the progression of cognitive deficits in stress-related pathologies. The underlying mechanisms for the augmented PSD are still unknown. However, it seems reasonable to admit that an imbalance in excitatory/inhibitory inputs is present. Several lines of evidence point on this direction. On one hand, stress exposure was shown to enhance the release of excitatory neurotransmitters (such as glutamate, dopamine, norepinephrine, or acetylcholine) in the hippocampus and PFC (Finlay et al., 1995; Mark et al., 1996; Feenstra, 2000; Moghaddam, 2002; Dazzi et al., 2004); on the other hand, it is known that GABAergic activity is reduced after stress exposure in these brain regions (Otero Losada, 1988; Hasler et al., 2010). Interestingly, once the increase in PSD is established in the vHIP, the changes in the mPFC might also be due to an augmented afferent excitatory input; in this case an early increase in the activation of hippocampal neurons could lead consequently to overactivation of monosynaptically connected (Jay and Witter, 1991; Thierry et al., 2000; Tierney et al., 2004) neurons in the mPFC. Such uncontrolled and desynchronized neuronal activity increase is likely to underlie the striking decrease in coherence between the vHIP and the mPFC observed after CUS, which may trigger a compensatory mechanism, translated by an increased power activity, in order to attempt to restore the decreased coherence in this neuronal network. This detrimental effect fits with the loss of important connections during neuronal atrophy verified in stressed subjects (Cerqueira et al., 2007a,b).

Coherence, as a measure of synchronization between the oscillations of cortical and limbic regions, was shown to underlie good behavior performance, namely supporting memory processing (Buzsáki, 1996, 2010; Jones and Wilson, 2005; Siapas et al., 2005; Benchenane et al., 2010; Popa et al., 2010; Hyman et al., 2011). In this scope, it is predictable that a decrease of coherence may cause impairments in the functions attributed to the regions that are connected by such circuits. Indeed, decreased coherence in the hippocampus-mPFC was shown to affect directly the PFC function (Brockmann et al., 2011). Based in the present observations, we suggest that the pronounced loss of vHIP-mPFC coherence between 1–40 Hz after chronic stress exposure underlie the previously described stress-related behavior impairments, which are dependent on the normal hippocampal and prefrontal function in those frequency bands (Cerqueira et al., 2007a). Importantly, the CUS-induced decrease in vHIP-mPFC coherence is associated with an enhancement of neuronal activity, measured by PSD, in coincident frequencies in both regions. The correlation of these two electrophysiological phenomena revealed an inverse correlation between vHIP-mPFC coherence and the hippocampal function, but not between mPFC activity and vHIP-mPFC coherence. This indicates a putative leading role of hippocampal firing on the disruption of this direct pathway by chronic stress exposure; the hippocampal disruption would, in turn, compromise the behavior outputs dependent on this pathway. In fact, these observations confirm previously reported behavioral data, in which reference memory (hippocampal-dependent) was impaired after 3 days of stress, while behavior flexibility (PFC-dependent) was only after a longer period of exposure to stress (Cerqueira et al., 2007a). Accordingly, this sequential behavior impairment is supported by a reduction of spine density and apical dendrite arborization of pyramidal neurons of layers II/III of the PFC, precisely where the hippocampal afferents establish synapses (Radley et al., 2004; Cerqueira et al., 2007b). Finally, it is noteworthy that after STS rats show a general increase in vHIP PSD, which is less extent in the mPFC of the same rats, and may indicate an early affection of the hippocampal neural activity by stress. Interestingly, in contrast to CUS, STS rats do not present yet signs of LTP impairment.

In addition, we confirmed that the decrease of vHIP-mPFC coherence and increase in PSD is linked to the poorer cognitive performance of stressed rats, as we found significant correlations between reference memory performance and electrophysiological data. This evidence is of major relevance since it establishes for the first time the link between the affection of the neural network after chronic stress exposure and the cognitive decline observed in the stressed subjects. Importantly, it confirms that coherence and neural activity, namely in the theta band, is severely affected in the stressed rats and is disruptive for the entrainment of the hippocampal-cortical connection and, consequently, for good behavior performance (Buzsáki, 1996; Jones and Wilson, 2005; Siapas et al., 2005; Benchenane et al., 2010; Popa et al., 2010; Hyman et al., 2011).

In summary, the present data reveals that chronic stress primarily affects hippocampal activity, which in turn will induce damage in prefrontal dendritic trees, disrupting the HIP-PFC connection and triggering a decrease in coherence between the two areas. Since neural coherence is a measure of regional interplay, behavior outcomes dependent on this interplay are severely affected, as showed by direct correlations between decrease in vHIP-mPFC coherence and reference memory performance. These findings provide a novel readout of the dynamics of stress deleterious effects in corticolimbic networks and are of relevance to better understand the mechanisms underlying stress-induced impairment in cognitive performance.

## Materials and methods

### Animals and treatments

All experiments were conducted in accordance with local regulations (European Union Directive 86/609/EEC) and National Institutes of Health guidelines on animal care and experimentation.

Two months-old male Wistar–Han rats (Charles River Laboratories, Spain) were housed in groups of two under standard laboratory conditions (lights on from 8:00 A.M. to 8:00 P.M.; room temperature 22°C; *ad libitum* access to food and water). A group of rats was submitted to 28 days of CUS. Briefly, rats were exposed to a stressor (1 h/day) of one of several aversive stimuli [cold water (18°C), restraint, overcrowding, exposure to a hot air stream, noise or shaking]; the stressors were presented in a random order. This stress paradigm was shown previously to induce persistently a pattern of consequences characterized by elevated plasma levels of corticosterone, the primary glucocorticoid of the rat, and reduced thymus weights (Cerqueira et al., 2007a). STS was induced to a second group of rats with one stressor daily (similar to CUS) for 6 days. A third group of rats was handled daily and served as controls (CON). Electrophysiological recordings were performed for each rat the day after the end of treatment.

An additional set of rats was used to search for correlations between behavioral performance and variations in power spectra in vHIP and mPFC and in coherence between these regions. Corticosterone levels were measured in blood serum sampled between 9:00 and 10:00 A.M. using a commercially available ELISA kit (Cayman Chemical, USA).

### Surgery

Rats were anesthetized with sodium pentobarbital (60 mg/kg, i.p., supplemented each 60 min throughout the experiment) and placed in a stereotaxic frame. Rectal temperature was maintained at 37°C by a homoeothermic blanket (Stoelting, Ireland). Experimental procedures for implantation and recording extracellular field potentials in the prelimbic area (PL) of the medial PFC have been described previously (Rocher et al., 2004; Cerqueira et al., 2007a). Briefly, Platinum/Iridium recording electrodes (Science Products, Germany) were placed in the PL (coordinates, 3.3 mm anterior to bregma, 0.8 mm lateral to the midline, 4.0 mm below bregma) and a concentric bipolar tungsten/stainless-steel electrode (WPI, USA) was positioned into the ipsilateral CA1/subicular region of the vHIP (coordinates, 6.5 mm posterior to bregma, 5.5 mm lateral to the midline, 5.3 mm below bregma), according to the atlas of Paxinos and Watson (2005).

### *In vivo* recording of local field potentials in the mPFC and ventral hippocampus

**Figure A4:**
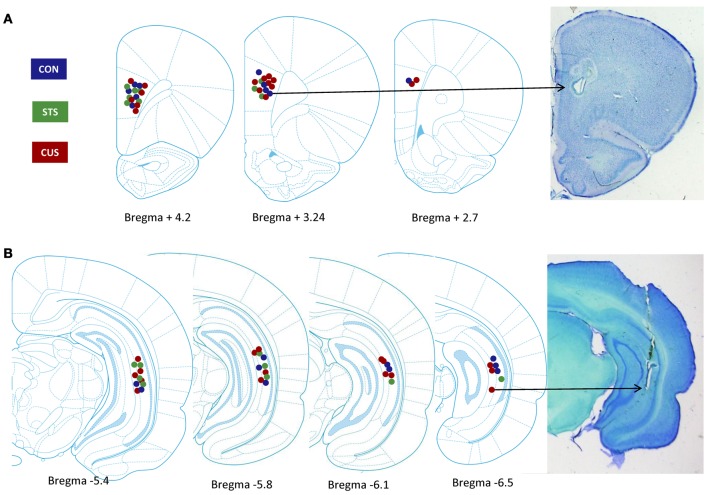
**Identification of electrode recording sites.** Left panel, recording site location in control (CON, blue), short term stress (STS, green) and chronic unpredictable stress (CUS, red) rats, within the medial prefrontal cortex (**A**, mPFC) and ventral hippocampus (**B**, vHIP); Images adapted from Paxinos and Watson, 2005. Right panel, Cresyl Violet stained 50 μm sections with an electrolytic lesion at the recording site.

LFP signals obtained from both electrodes were amplified, filtered (0.1–3000 Hz, LP511 Grass Amplifier, Astro-Med, Germany), acquired (Micro 1401 mkII, CED, UK) and recorded on a personal computer running Signal Software (CED, UK). After reaching fully anesthetized state, 100 s of local field activity was recorded at the sampling rate of 250 Hz (for representative traces see Figures 1A–D). After the electrophysiological protocols, a biphasic 1 mA stimulus was delivered to both electrodes. Rats were sacrificed and perfused a solution of paraformaldehyde 4%. Brains were carefully removed and sectioned in 50 μm slices. Brain slices containing the PFC and hippocampus were processed with a light Giemsa staining for determination of electrode position; the lesion caused by the biphasic stimulus identified the electrode position and the recordings were discarded whenever one of the electrodes failed the targeted position (about 12% of the recordings). In total we analyzed 6 CON, 9 STS, and 12 CUS rats; an additional set of 4 CON and 4 CUS were used to establish correlations between behavioral performance and electrophysiological data (Figure A4).

### *In vivo* study of synaptic plasticity between the mPFC and ventral hippocampus

Synaptic plasticity was tested in anesthetized rats by inducing LTP as described previously (Cerqueira et al., 2007a). Briefly, the electrode inserted in the CA1/subicular region was used to deliver stimuli to induce a characteristic monosynaptic field excitatory postsynaptic potential (fEPSP) in the PFC (Figure 1A; inset). Test pulses (100 ms) were delivered every 30 s at an intensity enough to evoke a potential about 70% of its maximum (250–500 μA; S88X Grass Stimulator, Astro-Med, Germany). The evoked potential to such stimulation is likely to reflect summated PSPs. Basal responses were recorded during 30 min and followed by LTP induction, which was obtained performing HSF, that consisted of two series of 10 trains (250 Hz, 200 ms) at 0.1 Hz, 6 min apart, delivered at test intensity. The size of LTP induction was measured by changes in the slope of responses to additional 90 min stimulating each 30 s. PSP slopes were analyzed using Signal software (CED, UK; sampling rate, 10 KHz) and expressed as a percentage change of the mean responses to basal stimulation before and after HFS.

### Behavioral analysis

Behavioral tests were conducted as described previously (Cerqueira et al., 2007a), using water maze-based tests to assess spatial reference memory and behavior flexibility. The spatial reference memory task, highly dependent of the integrity of the function of the hippocampus (Morris, 1984), assesses the ability of rats to learn the position of the hidden platform, based on external cues placed outside the pool. Rats were placed, facing the wall of the maze, in each of the four imaginary pool quadrants at the beginning of each of the four daily trials. A trial is considered complete when the rat escapes onto the platform; when this escape fails to occur within 120 s, the rat is gently guided to the platform and an escape latency of 120 s is recorded for that trial. Rats are allowed to spend 30 s on the escape platform before being positioned at a new starting point. Time spent to reach the platform (escape latency) was recorded in the consecutive trials. During the reference memory task, the platform was set in the same imaginary pool quadrant and rats were tested during 4 days. Data on average escape latencies to the platform on days 1–4 were analyzed as a reference memory test.

To perform the reversal learning task, a PFC-dependent function (De Bruin et al., 1994), on day 5, the escape platform was positioned in the opposite (new) quadrant and rats were tested in a 4-trial paradigm, similar to that described above. For this reverse-learning task, time spent swimming in the new quadrant were recorded and analyzed as a measure of behavioral flexibility.

## Data analysis and statistics

The PSD of PFC and hippocampus regions, as well as the coherence between both regions, were performed on the 2-channel 100 s long LFP signals acquired at the start of the experiment for each rat. Each measure was applied on 1 s long segments and the average of all segments was considered for statistical group analysis. Both measures were assessed in a wide range of frequencies: delta (1–4 Hz); theta (4–12 Hz); beta (12–20 Hz); low gamma (20–40 Hz); high gamma (40–90 Hz). All LFP records were thoroughly inspected and those that presented significant noise corruption were excluded from further analyses. PSD and coherence were calculated with custom-written MATLAB-based programs (MathWorks, Natick, MA).

### PSD analysis

The PSD of each channel was calculated through the 10 × log_10_ of the multiplication between the complex Fourier transform of each 1 s long data segment and its complex conjugate. The mean PSD of each channel was evaluated for all frequencies from 1 to 90 Hz.

### Spectral coherence analysis

Coherence analysis was based on multi-taper Fourier analysis. Coherence was calculated by custom-written MATLAB scripts, using the MATLAB toolbox Chronux (http://www.chronux.org) (Mitra and Pesaran, 1999). Coherence has been calculated for each 1 s long segments and their mean was evaluated for all frequencies from 1 to 90 Hz.

### Coherence-PSD correlations

The correlation values were calculated between the following paired frequency-domain vectors: coherence—mPFC PSD; coherence—vHIP PSD. The correlation measures were calculated through a custom-written MATLAB program that computes pairwise Pearson's coefficient between frequency-domain (1 Hz bins) coherence and PSD vector pairs. The *p*-values were calculated through a two-tailed *t*-test.

### Statistical group analysis

The group mean comparisons were calculated through non-parametric Mann–Whitney tests.

Multiple comparisons, for instance to compare LTP induction in the three groups (Figure A1), were made using non-parametric Kruskal–Wallis test followed by Dunn's corrections.

Performance of each group in the reference memory task and respective swim speeds were compared using repeated-measures ANOVA, being the difference between groups in each day calculated by Bonferroni *post-hoc* tests.

Pearson correlation coefficients were calculated between behavioral parameters and PSD, for both PFC and hippocampus regions, and vHIP-mPFC coherence.

### Conflict of interest statement

The authors declare that the research was conducted in the absence of any commercial or financial relationships that could be construed as a potential conflict of interest.
